# HIV‐associated Burkitt lymphoma in the combination antiretroviral therapy era: Real‐world outcomes and prognostication

**DOI:** 10.1002/jha2.624

**Published:** 2022-12-15

**Authors:** Chaoyu Wang, Shunsi Liang, Xi Quan, Bingling Guo, Dehong Huang, Jieping Li, Yao Liu

**Affiliations:** ^1^ Department of Hematology Oncology Chongqing University Cancer Hospital Chongqing Key Laboratory of Translational Research for Cancer Metastasis and Individualized Treatment Chongqing China

**Keywords:** Burkitt's lymphoma, clinical characteristics, HIV, outcomes, prognostication

## Abstract

We performed a retrospective study to analyze the clinical characteristics and outcomes of human immunodeficiency virus–associated Burkitt's lymphoma in Chongqing University Cancer Hospital, southwest China, from March 2012 to February 2022. In the entire cohort, the median age was 36 years (range, 28–60 years), and more patients were male (82.4%). The median CD4^+^ T cell count was 214/μl (range, 54–601), of whom 47.1% had a CD4^+^ T cell count below 200/μl. Most patients had elevated lactate dehydrogenase (LDH), elevated β_2_‐MG, extranodal involvement and advanced Ann Arbor stage at diagnosis. With a median follow‐up of 11.5 months (range, 1.6–94.9 months), the overall 1‐year progression‐free survival and overall survival (OS) rates were 27.6% and 47.6%, respectively. The 1‐year OS times in the LDH < 3 upper limit of normal and LDH ≥ 3 upper limit of normal groups were 62.5% and 31.3%, respectively (*p =* 0.008). The 1‐year OS times in the received <4 cycles and ≥4 cycles groups were 0% and 77.8%, respectively (*p <* 0.001). These results demonstrated that LDH < 3 upper limit of normal and received ≥4 cycles of chemotherapy were significantly associated with improved outcomes. However, rituximab administration was not significantly associated with improved outcomes.

## 1 INTRODUCTION

According to the different coincidence rate, human immunodeficiency virus (HIV)‐related tumors can be divided into HIV/acquired immune deficiency syndrome (AIDS)‐defining malignancies and non‐HIV/AIDS‐defining malignancies. HIV/AIDS ‐defining malignancies include Kaposi's sarcoma (KS), non‐Hodgkin's lymphoma (NHL), and invasive cervical cancer [1, 2]. Since 2017, the incidence of HIV‐associated NHL has been higher than that of KS, becoming the highest incidence rate among HIV‐associated cancers [2]. Burkitt's lymphoma (BL) is the second common subtype of HIV‐associated NHL after diffuse large B‐cell lymphoma (DLBCL) [3].

BL is a rare aggressive B‐cell NHL and is characterized by accelerated proliferation and cellular growth [4, 5, 6, 7]. Compared with HIV‐negative BL, most HIV‐positive BL have extra‐nodal involvement, advanced stage, and elevated lactate dehydrogenase (LDH) [8, 9]. Besides, the incidence of positive Epstein–Barr virus (EBV)‐encoded RNA (EBER) was 25%–40% in the HIV‐positive BL, and it was only 3%–5% in the HIV‐negative BL [10, 11].

So far, there are no standard guidelines for first‐line treatment of HIV‐associated BL. Studies regarding clinical characteristics and outcomes in HIV‐associated BL treated in the combination antiretroviral therapy (cART) era remain scarce. The optimal first‐line treatment regimens to improve survival are still not established. Chongqing University Cancer Hospital (CUCH) is the only cancer center in Western China that treats HIV‐associated lymphoma patients. Herein, we conducted a retrospective analysis to identify clinical characteristics and prognostic factors in HIV‐associated BL.

## 2 METHODS

### 2.1 Patients

All newly diagnosed patients, with HIV‐associated BL between March 2012 and February 2022 at CUCH, were retrospectively reviewed. The exclusion criteria were as follows: patients not received anti‐lymphoma therapy or lost follow‐up. Finally, a total of 17 HIV‐associated BLs were enrolled. The diagnosis was based on the 2008 World Health Organization (WHO) classification criteria. All patients were coinfected with HIV. This study was approved by the institutional review board of CUCH and conducted according to the Declaration of Helsinki. All patients signed the informed consent form.

### 2.2 Clinical data analysis

Clinical data, including demographics, HIV transmission route, time from HIV infection to lymphoma diagnosis, CD4 cell count at lymphoma diagnosis, Eastern Cooperative Oncology Group performance status, serum LDH, Ann Arbor stage, Age‐adjusted International Prognostic Index (aaIPI) score, extranodal involvement, bone marrow involvement, central nervous system (CNS) involvement, the presence of bulky tumor (maximum diameter ≥7.5 cm), and other related characteristics, including EBV load, EBER, and HBsAg, were analyzed.

### 2.3 Treatment and response assessment

The EPOCH regimen consisted of 96‐h continuous infusion of etoposide (50 mg/m^2^/day), doxorubicin (10 mg/m^2^/day), and vincristine (0.4 mg/m^2^/day), plus oral prednisone (60 mg/m^2^/day) from day 1 to day 5 and intravenous cyclophosphamide (375 mg/m^2^/day) on day 5, every 21 days. R‐EPOCH regime consisted of infusion of intravenous rituximab (375 mg/m^2^/day, prior to each chemotherapy), and 96‐h continuous infusion of etoposide (50 mg/m^2^/day), doxorubicin (10 mg/m^2^/day), and vincristine (0.4 mg/m^2^/day), plus oral prednisone (60 mg/m^2^/day) from day 1 to day 5 and intravenous cyclophosphamide (375 mg/m^2^/day) on day 5, every 21 days. A total of 11 patients received EPOCH regimen, and 6 patients received R‐EPOCH regime. The median number of cycles of chemotherapy was 4 (range, 1–14). All patients were administered cART. cART included two nucleoside reverse transcriptase inhibitors and one nonnucleoside reverse transcriptase inhibitor. All 17 patients were routinely administered CNS prophylaxis by intrathecal methotrexate, cytarabine, and dexamethasone.

Computed tomography (CT) or ^18^F‐fludeoxyglucose positron emission tomography/CT was performed for radiological evaluation. The 2007 revised Cheson criteria were used to define complete response (CR), partial response (PR), stable disease, and progressive disease (PD).

### 2.4 Statistical analysis

Progression‐free survival (PFS) was defined as the time from lymphoma diagnosis to disease progression, relapse, or death from any cause. Overall survival (OS) was defined as the time from lymphoma diagnosis to last follow‐up or death from any cause. All statistical data were analyzed with SPSS version 26 or GraphPad Prism 9. Survival was estimated using Kaplan–Meier curves and compared by the log‐rank test. *p* < 0.05 was considered statistically significant.

## 3 RESULTS

### 3.1 Patient characteristics

The baseline clinical features of these patients are summarized in Tables 1 and 2. There were no significant differences in each of the parameters between patients on EPOCH or R‐EPOCH besides β_2_‐MG. All the 17 patients had HIV before lymphoma was diagnosed. In addition, all the patients were on cART at diagnosis.

**TABLE 1:** Baseline clinical characteristics of patients

Baseline characteristics	Total *N* = 17 (%)	EPOCH *N* = 11 (%)	R‐EPOCH *N* = 6 (%)	*p*
Gender				0.938
Male	14 (82.4)	9 (81.8)	5 (83.3)	
Female	3 (17.6)	2 (18.2)	1 (16.7)	
Age, year				0.402
Median (range)	36 (28–60)	44 (32–60)	36 (31–50)	
<40	9 (52.9)	5 (45.5)	4 (66.7)	
≥40	8 (47.1)	6 (54.5)	2 (33.3)	
Ann Arbor stage				0.938
Early stage (I/II)	3 (17.6)	2 (18.2)	1 (16.7)	
Advanced stage (III/IV)	14 (82.4)	9 (81.8)	5 (83.3)	
aaIPI				0.171
0–1	5 (29.4)	5 (45.5)	0	
2–3	12 (70.6)	6 (54.5)	6 (100)	
ECOG PS				0.211
0–1	14 (82.4)	10 (90.9)	4 (66.7)	
2–4	3 (17.6)	1 (9.1)	2 (33.3)	
Elevated LDH	14 (82.4)	8 (72.7)	6 (100)	0.159
≥3 upper limit of normal	8 (47.1)	4 (36.4)	4 (66.7)	0.232
B symptoms	10 (58.8)	6 (54.5)	4 (66.7)	0.627
Elevated β_2_‐MG	10 (58.8)	4 (36.4)	6 (100)	0.011
Bone marrow involvement	4 (23.5)	3 (27.3)	1 (16.7)	0.622
>1 extranodal sites	11 (64.7)	6 (54.5)	5 (83.3)	0.235
Bulky tumor (maximum diameter ≥7.5 cm)	7 (41.2)	4 (36.4)	3 (50.0)	0.585
HIV transmission route				0.585
Heterosexual	7 (41.2)	4 (36.4)	3 (50.0)	
Homosexual	10 (58.8)	7 (63.6)	3 (50.0)	
CD4 cell count (per μl)				0.858
Median (range)	214 (54–601)	214 (157–601)	220 (54–369)	
<200	8 (47.1)	5 (45.5)	3 (50.0)	
≥200	9 (52.9)	6 (54.5)	3 (50.0)	
Ki‐67 > 90%	16 (94.1)	11 (100)	5 (83.3)	0.163
EBER positive	9 (52.9)	8 (72.7)	1 (16.7)	0.088
HBsAg positive	2 (11.8)	2 (18.2)	0 (0)	
Cycles of chemotherapy				0.402
<4	8 (47.1)	6 (54.5)	2 (33.3)	
≥4	9 (52.9)	5 (45.5)	4 (66.7)	

*Note*: The EPOCH regimen consisted of etoposide, doxorubicin, vincristine, prednisone, and cyclophosphamide. R‐EPOCH regime consisted of rituximab and EPOCH. The extranodal sites, including lung, gastrointestinal tract, kidney, liver, bone, skin, ureter, adrenal gland, peritoneum, and pancreas.

Abbreviations: aaIPI, Age‐adjusted International Prognostic Index; EBER: Epstein‐Barr virus (EBV)‐encoded RNA; ECOG, Eastern Cooperative Oncology Group; HIV, human immunodeficiency virus; LDH, lactate dehydrogenase; β_2_‐MG, β_2_‐microglobulin.

**TABLE 2:** Clinical characteristics and treatment regimes of the 17 patients

Patients	Gender	Age	Stage	aaIPI	CD4 cell count (×10 ^ 6/L)	LDH (U/L)	Time of diagnosis of BL	Treatment
1	Male	28	IVB	2	274	484	2013/7/10	EPOCH × 6 + HD‐MTX × 8
2	Male	31	IIA	1	195	1262	2021/1/16	R‐EPOCH × 4 + R‐MA × 3 + GDP × 3 + BR × 2
3	Male	31	IVB	2	214	1324	2021/12/2	EPOCH × 2
4	Male	32	IVB	3	345	1146	2012/3/26	CHOP × 2
5	Male	34	IVB	2	369	428	2014/8/15	R‐EPOCH × 6
6	Male	35	IIB	1	601	584	2017/11/26	EPOCH × 4 + DICE × 2
7	Male	36	IIIA	1	214	186	2014/10/1	EPOCH × 3 + GDP × 3
8	Female	36	IVB	2	245	604	2021/1/7	R‐EPOCH × 7
9	Male	36	IVB	3	141	1056	2021/2/1	R‐EPOCH × 8 + R‐DHAP + R‐MTX
10	Male	43	IVB	2	356	840	2022/2/16	R‐EPOCH × 3
11	Male	44	IVA	2	245	476	2015/7/10	EPOCH × 2
12	Female	47	IVB	2	157	1009	2021/12/2	EPOCH × 2
13	Male	50	IVB	2	169	1014	2017/6/1	EPOCH × 2
14	Male	50	IVB	3	54	1271	2021/8/30	R‐EPOCH × 2
15	Female	59	IIIA	1	183	165	2016/5/24	EPOCH × 6
16	Male	60	IIIA	1	162	164	2017/8/30	EPOCH × 4
17	Male	60	IIA	1	177	240	2018/4/1	EPOCH × 2

Abbreviations: aaIPI, Age‐adjusted International Prognostic Index; BL, Burkitt's lymphoma; LDH, lactate dehydrogenase

### 3.2 Treatment‐related mortality

The most common toxicities during treatment were myelosuppression, which included neutropenia and thrombocytopenia. Compared with HIV‐negative patients in our center, there was no significant increase in infection. In the R‐EPOCH cohort, three patients died of PD. In the EPOCH, total eight patients died, which included two patients died of infection and six died of PD. Besides the patient along with CNS disease present at diagnosis, the other patients had no CNS relapse.

### 3.3 Treatment efficacy and outcomes

**FIGURE 1:**
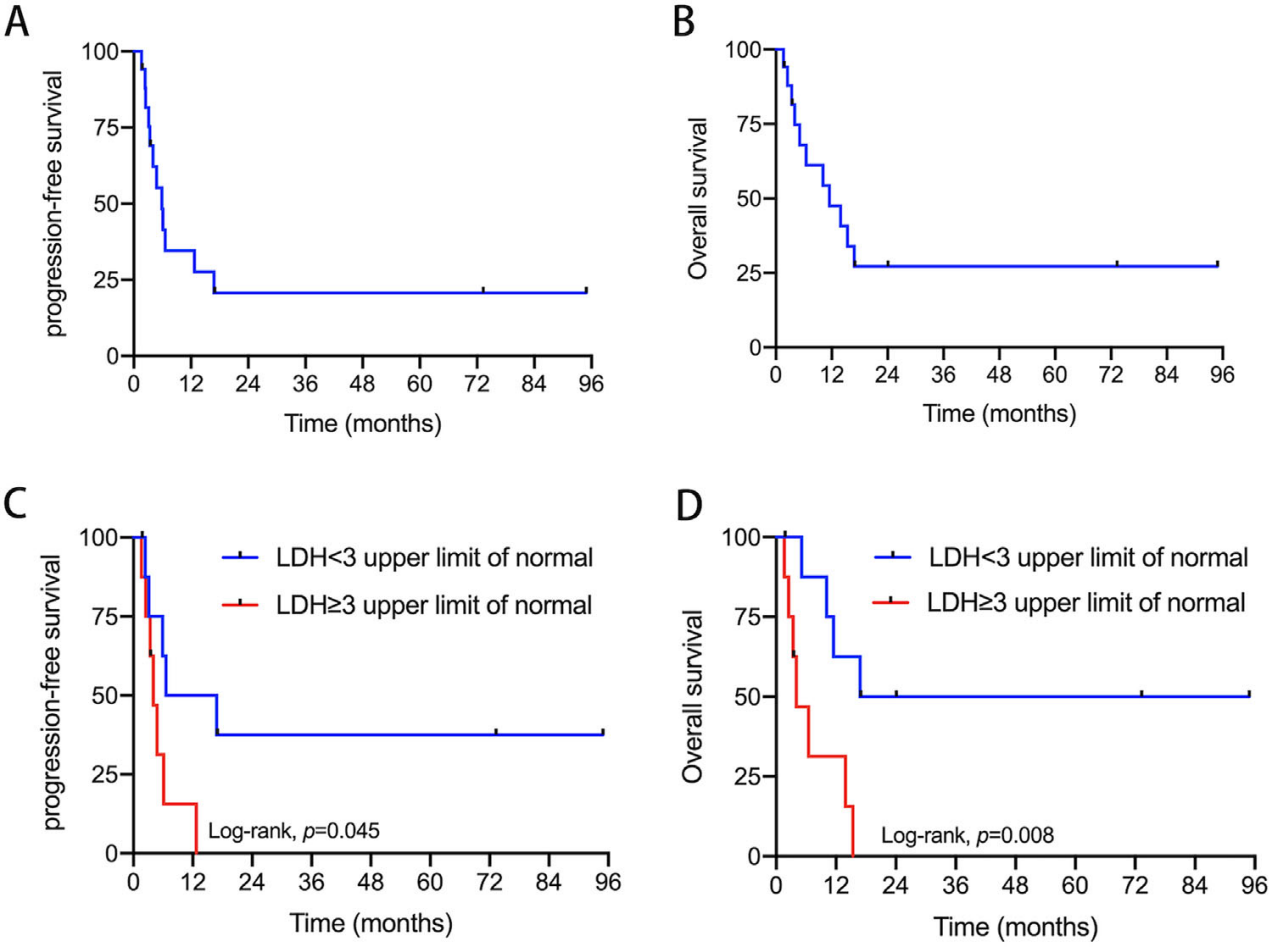
Survival for patients with human immunodeficiency virus (HIV)‐associated Burkitt's lymphoma enrolled in Chongqing University Cancer Hospital (CUCH): (A) Kaplan–Meier analysis of progression‐free survival (PFS) for all patients with HIV‐associated Burkitt's lymphoma; (B) Kaplan–Meier analysis of overall survival (OS) for all patients with HIV‐associated Burkitt's lymphoma; (C) PFS for patients stratified by lactate dehydrogenase (LDH); (D) OS for patients stratified by LDH

Among the 17 patients were evaluated for best treatment response at the end of treatment. Overall response rate (ORR) of 47.1% (8/17) includes two (11.8%) CR and six (35.3%) PR cases. Nine (52.9%) patients PD two patients in complete remission never relapsed and are alive now. Four patients out of six, who received PR after fist‐line chemotherapy, eventually relapsed, leading to death. So, the relapse rate was 50%. Of four relapse patients, two died after fist‐line chemotherapy, in which OS were 6.5 and 2.5 months. The other two patients all received second‐line chemotherapy, including R‐GDP and R‐DHAP, in which OS were 15.4 and 13.9 months. No patients receive bone marrow transplant or CAR‐T. With a median follow‐up of 11.5 months (range, 1.6–94.9 months), median PFS (Figure 1A) and OS (Figure 1B) were 5.9 and 11.5 months, respectively. The overall 1‐year PFS and OS rates were 27.6% and 47.6%, respectively.

**FIGURE 2:**
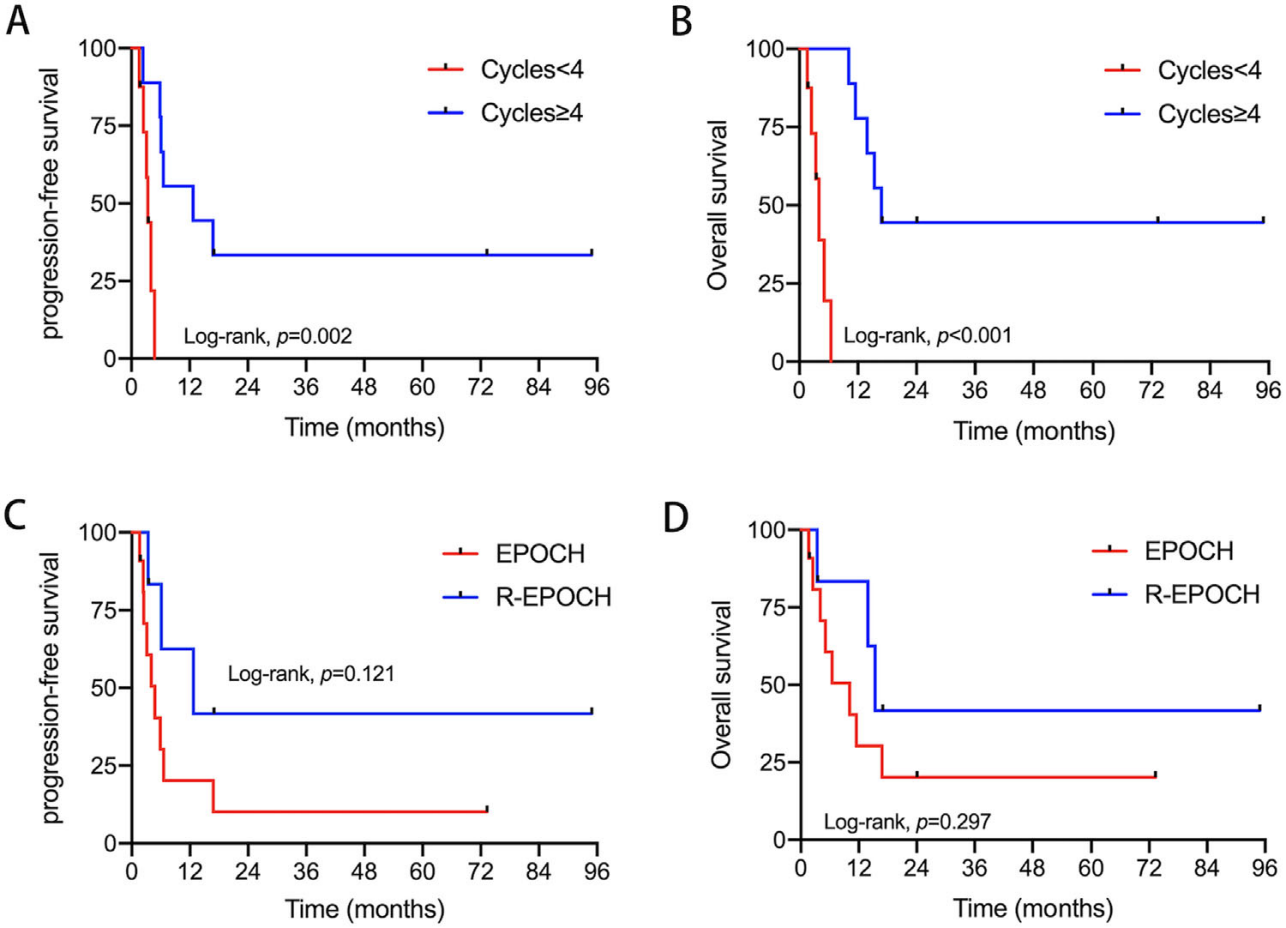
Kaplan–Meier analysis survival for patients with human immunodeficiency virus (HIV)‐associated Burkitt's lymphoma: (A) Kaplan–Meier analysis of progression‐free survival (PFS) for patients stratified by receiving different chemotherapy cycles; (B) Kaplan–Meier analysis of overall survival (OS) for patients stratified by receiving different chemotherapy cycles; (C) Kaplan–Meier analysis of PFS for patients stratified by receiving rituximab or not; (D) Kaplan–Meier analysis of OS for patients stratified by receiving rituximab or not

LDH represents the levels of LDH, and the upper limit of normal value is 245 U/L. The 1‐year PFS times in the LDH < 3 upper limit of normal and LDH ≥ 3 upper limit of normal groups were 50.0% and 15.6%, respectively (*p =* 0.045) (Figure 1C). The 1‐year OS times in the LDH < 3 upper limit of normal and LDH ≥ 3 upper limit of normal groups were 62.5% and 31.3%, respectively (*p =* 0.008) (Figure 1D). The 1‐year PFS times in the received <4 cycles and ≥4 cycles groups were 0% and 44.4%, respectively (*p =* 0.002) (Figure 2A). The 1‐year OS times in the received <4 cycles and ≥4 cycles groups were 0% and 77.8%, respectively (*p <* 0.001) (Figure 2B). These results demonstrated that LDH < 3 upper limit of normal and received ≥4 cycles of chemotherapy were significantly associated with improved outcomes. Other factors, including gender, age, stage, aaIPI, B symptoms, β_2_‐MG, extranodal involvement, bone marrow involvement, bulky tumor, and EBV coinfection, were not associated with patient prognosis (Table 3).

**TABLE 3:** Prognostic factor analysis for progression‐free and overall survivals

	PFS		OS	
Variables	*χ* ^2^	*p*‐Value	*χ* ^2^	*p*‐Value
Gender (female/male)	2.401	1.121	1.471	0.225
Age (<40/≥40)	0.001	0.990	0.400	0.527
Stage (I–II/III–IV)	1.072	0.301	0.085	0.770
aaIPI (0–1/2–3)	0.243	0.622	0.250	0.617
BL‐IPI (0/1/2–4)	2.077	0.354	5.544	0.063
B symptoms	1.717	0.190	1.222	0.269
ECOG (0–1/2–4)	1.057	0.304	3.315	0.069
CD4 (<200/≥200)	0.002	0.963	0.361	0.548
β_2_‐MG	1.136	0.287	0.946	0.331
LDH (<3*X*/≥3*X*)	3.665	0.045	6.966	0.008
CNS involvement	0.235	0.628	0.193	0.661
BM involvement	0.095	0.758	0.628	0.428
Extranodal	0.138	0.710	0.240	0.624
Bulky (>7.5 cm)	1.129	0.288	2.639	0.104
EBER (Neg/Pos)	0.128	0.720	0.144	0.704
Chemotherapy cycles (<4/≥4)	9.287	0.002	15.704	<0.001
Regimen (EPOCH/R‐EPOCH)	2.410	0.121	1.088	0.297

Abbreviations: aaIPI, Age‐adjusted International Prognostic Index; BL, Burkitt's lymphoma; CNS, central nervous system; EBER: Epstein–Barr virus (EBV)‐encoded RNA; ECOG, Eastern Cooperative Oncology Group; LDH, lactate dehydrogenase; OS, overall survival; PFS, progression‐free survival; β_2_‐MG, β_2_‐microglobulin

### 3.4 Rituximab administration and patient outcome

Of these patients with complete follow‐up data, 11 patients (64.7%) received EPOCH regimen, and 6 patients (35.3%) received R‐EPOCH regime. The overall 1‐year PFS rates for EPOCH and R‐EPOCH were 20.2% and 41.7%, respectively (*p =* 0.121) (Figure 2C); the overall 1‐year OS rates were 30.3% and 62.5%, respectively (*p =* 0.297) (Figure 2D). These data suggested that HIV‐associated Burkitt lymphoma patients receiving rituximab had higher OS, but more observations of HIV‐associated Burkitt lymphoma cases are needed to make a conclusive statement.

## 4 DISCUSSION

Little is known about the incidence and clinical features of HIV‐associated BL as these are less common than BL in the general population. These retrospective studies try to explain the prognostic factors and outcomes in HIV‐associated BL. Alderuccio [12] retrospectively analyzed 249 HIV‐associated BL from the United States and the United Kingdom at 35 centers from 2008 to 2019. The median age at diagnosis was 43 years (range, 23–77 years), and the majority of patients were male (84%). Most patients (85%) were elevated LDH, advanced stage (91%), and had extranodal (EN) involvement (60%). In our study, the median patient age at diagnosis was 36 years (range, 28–60 years), and 82.4% were male, 82.4% had elevated LDH, and most patients (64.7%) were advanced Ann Arbor stage at diagnosis. High aaIPI score (2–3) at diagnosis was found in 70.6% of patients. Overall, 64.7% had EN involvement. Overall, 41.1% patients showed bulky tumors, and 58.8% had B symptoms at diagnosis. These results demonstrated that HIV‐associated BL is more invasive.

Wang ES [13] demonstrated that HIV‐associated BL has similar CR rate and event‐free survival (EFS) rates compared to BL in general population when treated with a CODOX‐M/IVAC regimen. Montoto [14] analyzed 30 HIV‐associated BL, who were treated with CODOX‐M/IVAC, and found that 3‐year OS time was 52%. Cortes [15] enrolled 13 HIV‐associated BL and treated them with hyper‐CVAD/MA. The ORR was 100%, and 92% achieved CR. With a median follow‐up of 12 months, 2‐year OS time was 48%. Dunleavy [16] enrolled 30 newly diagnosed BL patients from 2000 to 2009, and 11 were positive for HIV. HIV‐associated BL underwent the SCEPOCH‐RR chemotherapy regimen, which is a lower dose short‐course combination with a double dose of rituximab. After a median follow‐up of 73 months, 100% of HIV‐associated BL patients had freedom from the progression of disease, and OS was 90%. Between 2010 and 2017, Roschewski [17] conducted a multicenter study of risk‐adapted DA‐EPOCH‐R in adults with untreated Burkitt lymphoma. The study totally enrolled 113 patients across 22 centers from the USA, and 28 (25%) were positive for HIV. At a median follow‐up of 58.7 months, EFS and OS were 84.5% and 87.0%, respectively. Therapy was equally effective across age groups, HIV status, and International Prognostic Index risk groups. So, Roschewski demonstrated that risk‐adapted DA‐EPOCH‐R therapy is effective in adult Burkitt lymphoma regardless of HIV status. In AMC 048 study [18], 13 AIDS Malignancy Consortium centers enrolled 34 HIV‐associated Burkitt lymphoma patients treated with cyclophosphamide, vincristine, doxorubicin, high‐dose methotrexate/ifosfamide, etoposide, and high‐dose cytarabine (CODOX‐M/IVAC) from 2007 to 2010. Most patients (94.1%) were high risk, 74% had advanced stage, and median CD4 count was 195 cells/ml (range, 0–721 cells/ml). The 1‐year progression‐free survival was 69% (95% confidence interval [CI], 51%–82%), and OS was 72% (95% CI, 53%–84%). Modifications of the CODOX‐M/IVAC regimen resulted in a grade 3–4 toxicity rate of 79%. Chamuleau [19] demonstrated that treatment with DA‐EPOCH‐R resulted in comparable CR and survival rates as R‐CODOX‐M/R‐IVAC but was associated with significantly less infectious complications, transfusions, and hospitalization days. This is the first multicenter randomized trial comparing two different chemotherapy regimens in patients with newly diagnosed BL. However, in our study, with a median follow‐up of 11.5 months (range, 1.6–94.9 months), the overall 1‐year OS rates were only 47.6%, which was significantly lower than that of other studies. The reason for the low survival rate may be related to insufficient intensity dose chemotherapy.

It is well known that rituximab can significantly improve the survival of BL in general population. Can the addition of rituximab further improve the survival of HIV‐positive BL? Barnes [20] enrolled 14 HIV‐positive BL patients, 10 patients received R‐CODOX‐M/IVAC as first‐line treatment regimen, and 4 patient received CODOX‐M/IVAC only. With a median follow‐up of 31.5 months (range 3.7–54.6 months), the 3‐year OS times were 77% and 66%, respectively (HR = 0.56, 95% CI 0.23–1.38; *p* = 0.43). Alderuccio [12] retrospectively analyzed 249 HIV‐associated BL. A total of 217 patients received rituximab‐containing regimen, and 32 patients did not receive rituximab. With a median follow‐up of 4.5 years, the 3‐year PFS times were 63% and 53% in the patients treated with rituximab‐containing regimen group and not received rituximab group, respectively (*p* = 0.17). In addition, the 3‐year OS times were 66% and 62%, respectively (*p* = 0.41). In our study, 1‐year OS times were 62.5% and 30.3%, respectively (*p =* 0.297). HIV‐associated BL receiving rituximab had a higher OS, but the difference did not reach statistical significance. These data suggested that rituximab administration was not significantly associated with improved outcomes in patients with HIV‐associated BL. In this study, HIV‐associated BL receiving rituximab had higher OS, but more observations of HIV‐associated BL cases are needed to make a conclusive statement.

In this study, we also found that LDH ≥ 3 upper limit of normal at diagnosis and received <4 cycles of chemotherapy were identified as poor prognostic factors associated with a higher risk of treatment failure, and HIV features no longer influence prognosis in HIV‐associated BL. The mechanism may be related to gluconeogenesis and DNA metabolism, as an LDH level serves as an important checkpoint of gluconeogenesis and DNA metabolism. Besides, we also found that HIV‐associated features, which include CD4 cell count did not affect prognosis of HIV‐associated BL. Habbous et al. [21] also found that HIV status did not affect prognosis for patients with DLBCL receiving R‐CHOP in a general population.

In summary, we found that HIV‐associated features no longer influence outcomes in HIV‐associated BL and also found that LDH ≥ 3 upper limit of normal at diagnosis and received <4 cycles of chemotherapy were the two prognostic factors associated with inferior survival. Currently, people with HIV positive are mostly excluded from clinical trials precluding the evaluation of novel agents. Hope more prospective clinical studies toward incorporating HIV‐positive BL in the future. Moreover, more prospective clinical studies are needed to establish a prognostic risk assessment system for HIV‐associated BL.

## AUTHOR CONTRIBUTIONS

*Conceived and designed the study; analyzed the data; and drafted and revised the paper*: Chaoyu Wang, Shunsi Liang, Jieping Li. *Conceptualized and designed the study*: Yao Liu. All authors provided critical comments to the manuscript. All authors contributed to the article and approved the submitted version.

## CONFLICT OF INTEREST

The authors declare that they have no conflicts of interest.

## ETHICAL STATEMENT

All procedures performed in studies involving human participants were in accordance with the ethical standards of the institutional and/or national research committee and with the 1964 Helsinki Declaration and its later amendments or comparable ethical standards.

## PATIENT CONSENT STATEMENT

All patients signed the informed consent form.

## Data Availability

The raw data supporting the conclusions of this article will be made available by the authors, without undue reservation.
